# 5-aminolevulinic acid combined with ferrous ion reduces adiposity and improves glucose tolerance in diet-induced obese mice via enhancing mitochondrial function

**DOI:** 10.1186/s40360-016-0108-3

**Published:** 2017-01-30

**Authors:** Urara Ota, Takeshi Hara, Hitoshi Nakagawa, Emi Tsuru, Masayuki Tsuda, Atsuko Kamiya, Yasushi Kuroda, Yuya Kitajima, Aya Koda, Masahiro Ishizuka, Hideo Fukuhara, Keiji Inoue, Taro Shuin, Motowo Nakajima, Tohru Tanaka

**Affiliations:** 1 SBI Pharmaceuticals Co. Ltd., 1-6-1, Roppongi, Minato-ku, Tokyo, 106-6020 Japan; 2 Institute for Laboratory Animal Research, Kochi Medical School, Kochi University, Kohasu, Oko-cho, Nankoku, 783-8505 Japan; 3 Department of Urology, Kochi Medical School, Kochi University, Kohasu, Oko-cho, Nankoku, 783-8505 Japan

**Keywords:** 5-aminolevulinic acid, Diet-induced obese mice, Glucose tolerance, Glucose uptake, Mitochondrial oxidative phosphorylation complex, Obesity, White adipose tissue

## Abstract

**Background:**

Mitochondrial dysfunction is associated with obesity and various obesity-associated pathological conditions including glucose intolerance. 5-Aminolevulinic acid (ALA), a precursor of heme metabolites, is a natural amino acid synthesized in the mitochondria, and various types of cytochromes containing heme contribute to aerobic energy metabolism. Thus, ALA might have beneficial effects on the reduction of adiposity and improvement of glucose tolerance through its promotion of heme synthesis. In the present study, we investigated the effects of ALA combined with sodium ferrous citrate (SFC) on obesity and glucose intolerance in diet-induced obese mice.

**Methods:**

We used 20-weeks-old male C57BL/6J diet-induced obesity (DIO) mice that had been fed high-fat diet from 4th week or wild-type C57BL/6J mice. The DIO mice were orally administered ALA combined with SFC (ALA/SFC) for 6 weeks. At the 4th and 5th week during ALA/SFC administration, mice were fasted for 5 h and overnight, respectively and used for oral glucose tolerance test. After the ALA/SFC administration, the plasma glucose levels, weight of white adipose tissue, and expression levels of mitochondrial oxidative phosphorylation (OXPHOS) complexes were examined. Furthermore, the effects of ALA/SFC on lipid content and glucose uptake were examined in vitro.

**Results:**

Oral administration of ALA/SFC for 6 weeks reduced the body weight by about 10% and the weight of white adipose tissues in these animals. In vitro, ALA/SFC reduced lipid content in mouse 3T3-L1 adipocytes in a dose dependent manner, and enhanced glucose uptake in 3T3-L1 adipocytes by 70–90% and rat L6 myoblasts by 30% at 6 h. Additionally, oral administration of ALA/SFC reduced plasma glucose levels and improved glucose tolerance in DIO mice. Furthermore, ALA/SFC enhanced the expression of OXPHOS complexes III, IV, and V by 40–70% in white adipose tissues of DIO mice, improving mitochondrial function.

**Conclusions:**

Our findings indicate that ALA/SFC is effective in the reduction of adiposity and improvement of glucose tolerance, and that the induction of mitochondrial OXPHOS complex III, IV, and V by ALA/SFC might be an essential component of the molecular mechanisms underlying these effects. ALA/SFC might be a useful supplement for obesity and obesity-related metabolic disease such as type 2 diabetes mellitus.

**Electronic supplementary material:**

The online version of this article (doi:10.1186/s40360-016-0108-3) contains supplementary material, which is available to authorized users.

## Background

Obesity is characterized as an expansion of adipose tissue mass; in particular, white adipose tissue (WAT). The incidence of obesity is rapidly increasing worldwide and represents a major global health problem [1, 2]. WAT has an important role in energy storage in the form of triglycerides and is known as an endocrine organ that secretes adipokines such as adiponectin and leptin [1, 3, 4]. It is believed that the imbalance between energy intake and expenditure accompanying a sedentary lifestyle and a diet rich in fats and sugar leads to the development of obesity [2, 5]. In turn, obesity is a risk factor for various pathological conditions such as glucose intolerance, hypertension, and hyperlipidemia [6]. Additionally, obesity is a major contributor to its associated diseases including type 2 diabetes mellitus (T2DM), cardiovascular disease, and certain cancers [1–3, 6–8], with central/visceral obesity being most closely linked to the development of these diseases [7, 8].

Mitochondria play essential roles in energy metabolism such as the production of ATP via oxidative phosphorylation (OXPHOS), generation of numerous metabolites by the tricarboxylic acid cycle, and β-oxidation [3, 9]. Mitochondrial dysfunction is associated with obesity [3, 9] and obesity-associated diseases [3, 10]. Caloric restriction and exercise training are important for stimulating mitochondrial activity, and have metabolic health effects [11, 12]. Compounds that activate mitochondrial function might also be useful for the improvement of obesity and its associated pathological conditions such as high blood pressure and hyperglycemia.

**Fig. 1:**
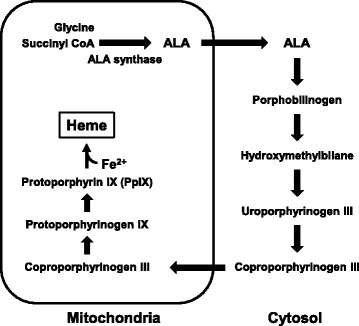
Heme synthesis pathway in animal cells. ALA is synthesized by the condensation of glycine and succinyl CoA via mitochondrial ALA synthase. The polymerization of 8 molecules of ALA in several subsequent steps produces protoporphyrin IX (PpIX). Heme is synthesized by the insertion of a ferrous ion into PpIX, and incorporated into proteins to produce heme-proteins such as hemoglobin, cytochrome, and P450

5-Aminolevulinic acid (ALA) is an endogenous amino acid that is widely distributed in both animals and plants [13]. In animal cells, ALA is synthesized by the condensation of glycine and succinyl CoA via mitochondrial ALA synthase (Fig. 1) [13]. In turn, heme is synthesized by the insertion of a ferrous ion into protoporphyrin IX (PpIX), which is generated from ALA in several subsequent steps [13], and incorporated into proteins to produce heme-proteins. These play important roles in the energy generating function of mitochondria [13, 14]. Heme deficiency has been suggested to decrease the protein expression and the activity of cytochrome *c* oxidase (complex IV) [15], which cause mitochondrial dysfunction [16] and might contribute to their decay with aging [17]. On the other hand, recent studies have reported that ALA upregulates aerobic energy metabolism through increasing the activity and protein expression of complex IV in the mitochondria of normal mice [18, 19]. Thus, ALA might be useful for reducing adiposity and blood glucose levels and an improvement of glucose tolerance through enhancing mitochondrial activity.

In the present study, we examined the effects of ALA combined with sodium ferrous citrate (ALA/SFC) on the reduction of adiposity and improvement of glucose tolerance in diet-induced obesity (DIO) C57BL/6J mice, a well-known animal model that mimics the human metabolic abnormalities observed in obesity [20], because ALA combined with ferrous ions enhances heme production [21–23]. We also examined the effects of ALA/SFC on lipid content and glucose uptake in cultured cells, and determined the expression levels of mitochondrial OXPHOS complex proteins in WAT of DIO mice.

## Methods

### Regents

5-Aminolevulinic acid hydrochloride (Lot number: HCL-KK08-04-1-1, purity; 99.8%) was obtained from Cosmo Oil Co., Ltd, (Tokyo, Japan). SFC was purchased from Komatsuya Corporation (Osaka, Japan). DL-Dithiothreitol, Oil Red O, sodium orthovanadate (Na_3_VO_4_), Tween 20, Eagle’s minimal essential medium (EMEM), and 100 × antibiotic antimycotic solution (ABAM) were purchased from Sigma-Aldrich Japan (Tokyo, Japan). Sodium fluoride (NaF), sodium deoxycholate, l-ascorbic acid phosphate magnesium salt n-hydrate, isopropanol, and Dulbecco’s modified Eagle’s medium (DMEM) with 4500 mg/L glucose (DMEM-high glucose) and 1000 mg/L glucose (DMEM-low glucose) were purchased from Wako Pure Chemicals (Tokyo, Japan). Fetal bovine serum (FBS) and Calf bovine serum (CBS) were purchased from BioWest (Nuaillé, France) and the American Type Culture Collection (ATCC, Manassas, VA, USA), respectively. Insulin (10 μg/mL human recombinant) was purchased from Life Technologies Japan (Tokyo, Japan). Halt Protease Inhibitor Cocktail was purchased from Thermo-Scientific (Waltham, MA, USA). High-fat diet (HFD) (60 kcal% fat, D12492) was purchased from Research Diet Inc. (New Brunswick, NJ, USA).

### Mice

Eighteen-weeks-old male C57BL/6J DIO mice fed HFD from 4th week and wild-type C57BL/6J mice were obtained from Charles River Japan (Yokohama, Japan). During the acclimation and ALA/SFC administration periods, C57BL/6J DIO mice were fed HFD while C57BL/6J mice were fed normal diet (ND), respectively. The mice were given HFD + vehicle, HFD + ALA/SFC, and ND + vehicle with 5, 5, and 8 animals in each group, respectively. All mice survived for the duration of the ALA/SFC administration.

### ALA/SFC administration

After 2-weeks acclimation, the 20-weeks-old C57BL/6J DIO or C57BL/6J mice were orally administered ALA/SFC or vehicle by gavage once per day for 6 weeks. The molar ratio of ALA to ferrous ion was 1 to 0.05. The dosing volume was 10 mL/kg.

### Oral glucose tolerance test (OGTT)

At the 4th and 5th week during ALA/SFC administration, mice were fasted for 5 h and overnight, respectively and used for OGTT. Blood samples were collected from the tail vein at 15, 30, 60, 90, and 120 min after oral administration of glucose (2 g glucose/10 mL/kg).

### Biochemical analysis

The blood samples were centrifuged at room temperature at 800 × *g* for 5 min, and the hematocyte fractions were used for measurement of HbA1c levels via the Norudia N HbA1c enzymatic method (Sekisui Medical Co., Ltd., Tokyo, Japan). The remaining supernatants were further centrifuged at room temperature at 2000 × *g* for 10 min and the resultant supernatants were used for measurements of plasma glucose and insulin levels. The plasma glucose level was determined by the glucose oxidase method with CicaLiquid GLU (Kanto Chemical, Tokyo, Japan). The plasma insulin level was determined using a rat insulin ELISA kit (Morinaga Institute of Biological Science, Inc., Kanagawa, Japan).

### Cell culture

The mouse embryonic fibroblast line 3T3-L1 (ATCC CL-173) was obtained from the ATCC. Rat skeletal L6 myoblasts (ATCC IFO50364) were provided by the Japanese Collection of Research Bioresources Cell Bank (Osaka Japan). All cells were cultured at 37 °C in a 5% CO_2_ atmosphere. 3T3-L1 mouse embryonic fibroblasts were routinely cultured in DMEM-high glucose supplemented with 10% CBS and 1% 100 × ABAM at 37 °C. Adipocyte differentiation was induced as previously reported [24]. Briefly, confluent grown 3T3-L1 cells were shifted into adipocyte differentiation medium (DMEM-high glucose supplemented with 10% FBS, 1% ABAM (100 ×), 10 μg/mL insulin, 1.0 μM dexamethasone, 0.5 mM methylisobutylxanthine, and 100 μM l-ascorbic acid phosphate magnesium salt n-hydrate). After 48 h, the medium was changed to adipocyte maintenance medium (DMEM-high glucose with 10% FBS and 1% ABAM (100 ×) plus 10 μg/mL insulin and 100 μM l-ascorbic acid phosphate magnesium salt n-hydrate). The adipocyte maintenance medium was renewed every 2 to 3 days. After 7 to 14 days, the differentiated 3T3-L1 adipocytes were used for the experiments.

Myotube differentiation was induced as previously reported [25]. Briefly, L6 myoblasts were maintained in EMEM supplemented with 10% FBS and 1% ABAM. At confluence, L6 myoblasts were shifted to differentiation medium (EMEM supplemented with 2% FBS and 1% ABAM (100 ×)) to induce myotube differentiation. The differentiation medium was exchanged every 2–3 days. At 7–10 days after induction, the differentiated L6 myotubes were used for the experiments.

### Oil Red O staining

Oil Red O staining of neutral triglycerides and lipids and the determination of its concentration were performed as reported previously with slight modification [26]. Briefly, differentiated 3T3-L1 adipocytes were washed twice with PBS, fixed in 10% formaldehyde for 10 min, rinsed with 60% isopropanol for 1 min, and stained with the Oil Red O solution in 60% isopropanol for 20 min. After staining, the adipocytes were washed twice with PBS and observed under a microscope. Oil red O was extracted from the cells with 100% DMSO and quantified by its absorbance at 540 nm using an Infinite M200 PRO multiple plate reader (Tecan, Männedorf, Switzerland).

### Measurement of 2-deoxyglucose (2DG) uptake

Uptake of 2DG was measured using a 2DG Uptake Measurement kit (Cosmo Bio, Tokyo, Japan) according to manufacturer’s procedure [27]. Briefly, cells were cultured in 12-well plates for 18 h in DMEM containing 10% FBS with or without ALA/SFC and incubated for 6 h in DMEM containing 0.1% BSA with or without ALA/SFC. For treatment with ALA or SFC only, the cells were incubated with MEM containing 0.1% BSA. Next, the cells were incubated in Krebs-Ringer’s phosphate (KRPH) buffer (20 mM HEPES, 137 mM NaCl, 4.7 mM KCl, 1 mM MgSO_4_, 5 mM KH_2_PO_4_, 1 mM CaCl_2_, and 2 mM pyruvate, pH 7.4) for 18 min at 37 °C. The cells were further incubated with KRPH buffer containing 1 mM 2DG with or without insulin for 20 min and then lysed by sonication. Protein concentrations were determined by Pierce™ BCA Protein Assay Kit (Thermo Fisher Scientific, Rockford, IL, USA).

### Western blotting analysis

Epididymal fat was lysed in lysis buffer (10 mM Tris–HCl, pH 8.0, 150 mM NaCl, 1.0% NP-40, 0.5% sodium deoxycholate, 0.1% SDS and 1 mM DTT) containing 1% Halt Protease Inhibitor Cocktail (100×) and phosphatase inhibitors (10 mM NaF and 1 mM Na_3_VO_4_) for 1 h on ice [28]. After centrifugation (20,000 × *g*, 20 min, 4 °C), the supernatant was diluted with SDS sample buffer, fractionated using 7.5–10% gradient SDS-PAGE, and electrotransferred onto Immobilon-P PVDF membranes (IPVH07850, Merck Millipore, Billerica, MA, USA).

Detection of mitochondrial complexes I to V was performed as follows: the membranes were treated with 1% BSA in TBST (50 mM Tris-HCl pH7.6, 150 mM NaCl, and 0.05% Tween 20) for 1 h at room temperature, followed by overnight incubation with MitoProfile® Total OXPHOS Rodent WB Antibody Cocktail (dilution 1:1000) (ab110413, Abcam, Cambridge, UK) at 4 °C. After incubation, the membranes were washed five times for 10 min with TBST, incubated with an HRP-linked anti-mouse IgG antibody (dilution 1:10,000) (NA931, GE healthcare, NJ, USA) as the secondary antibody, washed five times with TBST for 10 min, and visualized with an ECL prime western blotting detection kit (RPN2232, GE Healthcare, Piscataway, NJ, USA) using a ChemiDoc MP system (Bio-Rad). For detection of β-actin, the membranes were blocked with 5% non-fat milk for 1 h at room temperature, and incubated with anti-β-actin antibody (dilution 1:5000) (ab8227, Abcam) at 4 °C overnight. An HRP-linked anti-rabbit IgG antibody (dilution 1:10,000) (NA934, GE Healthcare) was used as the secondary antibody; the specific protein signal was visualized as described above.

### Statistical analysis

The results were expressed as means ± standard deviation (SD). For mouse studies, statistical significance was determined the using a two-tailed unpaired Student’s *t*-test or one-way analysis of variance followed by Dunnett’s test. For in vitro studies, statistical significance was determined by one-way analysis of variance followed by Dunnett’s or Bonferroni’s tests. *p*-values less than 0.05 were considered statistically significant.

## Results

### ALA/SFC reduces adiposity in DIO mice

**Fig. 2:**
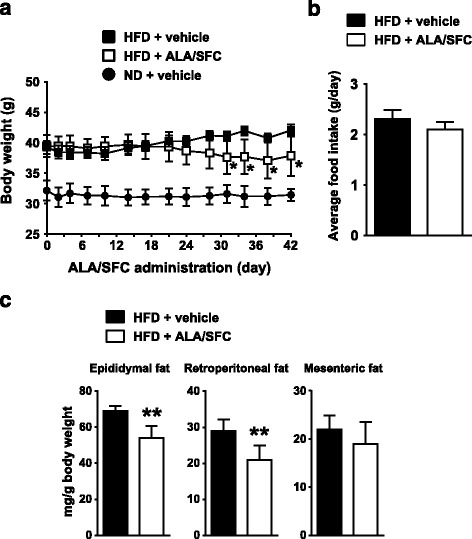
ALA/SFC reduces adiposity in DIO mice. DIO mice were orally administered ALA/SFC for 6 weeks. The numbers of mice in HFD + vehicle, HFD + ALA/SFC, and ND + vehicle groups were 5, 5, and 8, respectively. **a** Body weight. *p < 0.05 vs. vehicle-administered DIO mice (Student’s *t*-test). **b** Food intake (Student’s *t*-test). **c** Weights of epididymal, retroperitoneal, and mesenteric fat tissues normalized by body weight. **p < 0.01 vs. vehicle-administered DIO mice (Student’s *t*-test)

We first investigated the body weight changes in DIO mice following ALA/SFC treatment. As shown in Fig. 2a, the body weight of HFD-fed mice was higher than that of ND-fed mice, consistent with previous reports [29, 30]. There were no obvious clinical abnormalities following ALA/SFC administration. Until about 24 days from the initiation of administration, the average body weight of the ALA/SFC-administered DIO mice was not significantly different from that of the vehicle-administered DIO mice (Fig. 2a). However, from 31 to 42 days after administration, the body weight of ALA/SFC-administered DIO mice was significantly reduced by about 10% compared with vehicle-administered DIO mice. The average food intake of the ALA/SFC-administered DIO mice from initiation to day 42 was almost same levels compared with vehicle-administrated DIO mice (Fig. 2b).

An increase of body weight is reported to be associated with an increased weight of fat tissue including WAT in DIO mice [30, 31]. The reduced body weight of ALA/SFC-administered mice might be caused by a reduction of WAT weight. To confirm this, we measured the weight of WAT, epididymal, retroperitoneal, and mesenteric fat in DIO mice after ALA/SFC administration (Fig. 2c). The weights of epididymal and retroperitoneal fat in ALA/SFC-administered DIO mice were significantly reduced by 21 and 28%, respectively, compared with those in vehicle-administered DIO mice, whereas the weight reduction of mesenteric fat in the ALA/SFC-administered mice was moderate (a 12% reduction), and the difference was not significant. Collectively, these data suggest that ALA/SFC reduces adiposity in DIO mice.

We also measured the plasma concentrations of the triglycerides and total cholesterol after ALA/SFC administration. In vehicle-administered DIO mice, these were 66 ± 12 mg/dL and 169 ± 18 mg/dL (means ± SD, *n* = 5), respectively, whereas the concentrations in ALA/SFC administered DIO mice were 107 ± 53 mg/dL and 158 ± 20 mg/dL (*n* = 5), which were not significantly different from those in vehicle-administered DIO mice (Student’s *t*-test). These data suggest that ALA/SFC does not affect the plasma concentrations of triglycerol and total cholesterol in DIO mice.

### ALA/SFC reduces lipid content in 3T3-L1 adipocytes

**Fig. 3:**
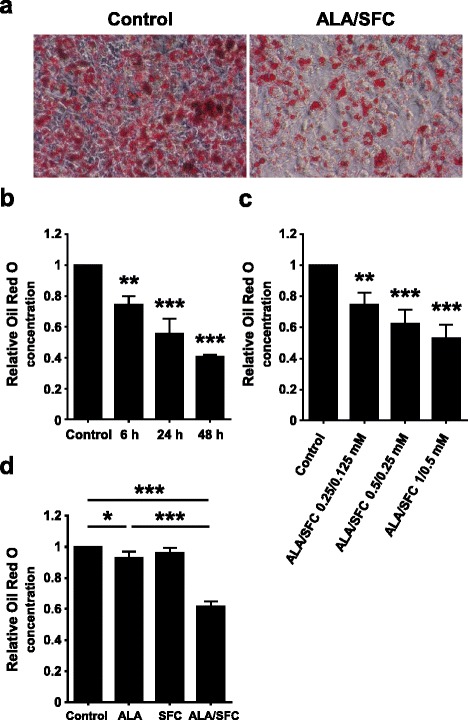
ALA/SFC reduces lipid content in 3T3-L1 adipocytes. 3T3-L1 adipocytes were cultured in adipocyte maintenance medium with or without ALA/SFC for the indicated times, stained with Oil Red O, and the extracted Oil red O was quantified by its absorbance at 540 nm. Results are shown as means ± SD relative to the control (set to 1.0). **a** Cell staining was observed after ALA (1 mM)/SFC (0.5 mM) treatment for 24 h. Representative figures are shown (original magnification, × 400). **b** Time-dependent effects of ALA/SFC. The cells were treated with ALA (1 mM)/SFC (0.5 mM) for the indicated times and then stained with Oil Red O. **p < 0.01, ***p < 0.001 vs. control (Dunnett’s test, *n* = 3). **c** Effects of 24-h treatment of various ALA/SFC concentrations on lipid content. **p < 0.01, ***p < 0.001 vs. control (Dunnett’s test, *n* = 4). **d** Effects of 24-h treatments with ALA (1 mM) alone, SFC (0.5 mM) alone, or ALA (1 mM)/SFC (0.5 mM) in combination on lipid content. *p < 0.05, ***p < 0.001 vs. control (Bonferroni’s test, *n* = 4)

To more precisely determine whether ALA/SFC might directly influence the reduction of adiposity in DIO mice, we next examined the lipid content in 3T3-L1 adipocytes after ALA/SFC treatment. Treatment of 3T3-L1 adipocytes with ALA/SFC did not induce cytotoxic effect under our conditions. As shown in Fig. 3a, 3T3-L1 adipocytes treated with ALA/SFC exhibited a reduction of lipid droplets compared to control cells. Furthermore, the lipid content in 3T3-L1 cells was reduced time- and dose-dependently after ALA/SFC treatment (Fig. 3b, c). This reduction was also observed in 3T3-L1 cells treated with ALA alone by 7% (Fig. 3d). However, the reduction of lipid content following ALA treatment alone was weaker than that from ALA/SFC treatment (Fig. 3d). In contrast, treatment with SFC alone did not reduce lipid content. Thus, these data suggest that ALA combined with ferrous ion reduces lipid content in 3T3-L1 adipocytes.

### ALA/SFC improves glucose tolerance in DIO mice

**Fig. 4:**
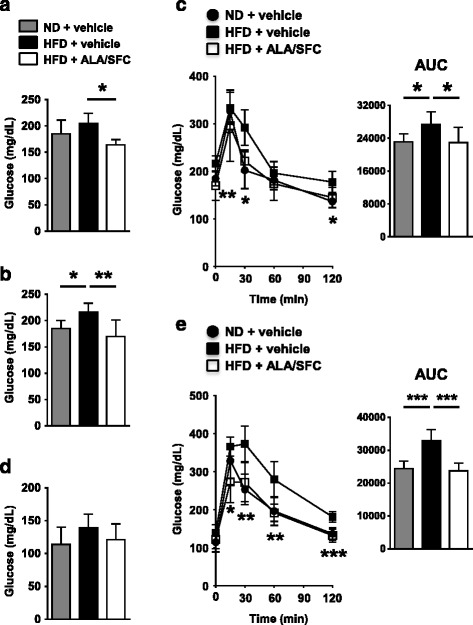
ALA/SFC improves glucose tolerance. DIO mice were orally administered ALA/SFC for 6 weeks. The numbers of mice in HFD + vehicle, HFD + ALA/SFC, and ND + vehicle groups were 5, 5, and 8, respectively. **a** Plasma glucose levels after 6-weeks administration of ALA/SFC. *p < 0.05, **p < 0.01 vs. vehicle-administered DIO mice (Dunnett’s test). **b**, **c** At 4th week after ALA/SFC administration, mice were fasted for 5 h and then OGTT was performed. **b** Fasting plasma glucose levels. **c** Plasma glucose levels and AUC. *p < 0.05, **p < 0.01 vs. vehicle-administered DIO mice (Dunnett’s test). **d**, **e** At 5th week of ALA/SFC administration, mice were fasted overnight and then OGTT was performed. **d** Fasting plasma glucose levels. **e** Plasma glucose levels and AUC. *p < 0.05, **p < 0.01, ***p < 0.001 vs. vehicle-administered DIO mice (Dunnett’s test)

Obesity is linked to increased plasma glucose levels and glucose intolerance. Thus, the plasma glucose levels were investigated in DIO mice after ALA/SFC administration. At 6th week under non-fasting conditions, the plasma glucose levels in ALA/SFC-administered DIO mice were significantly decreased by 20% compared with those in vehicle-administered DIO mice (Fig. 4a).

We twice performed OGTT to confirm the improvement of glucose tolerance following ALA/SFC administration in DIO mice. Mice were fasted for 5 h at 4th week and overnight at 5th week after ALA/SFC administration. The plasma glucose levels from 5 h fasting in ALA/SFC-administered DIO mice were 21%, significantly lower than those in vehicle-administered DIO mice (Fig. 4b). After glucose administration, ALA/SFC improved the glucose tolerance of DIO mice compared with vehicle-administered DIO mice (Fig. 4c). By OGTT, the glucose concentrations in plasma were almost the same between ALA/SFC-administered DIO mice and ND fed mice. In ALA/SFC-administered DIO mice, integrated glucose concentrations, as calculated by the area under the curve (AUC), were 84%, significantly decreased compared with vehicle-administered DIO mice (Fig. 4c). Although the plasma glucose levels following overnight fasting in ALA/SFC-administered DIO mice were not significantly different from those in vehicle-administered DIO mice (Fig. 4d), ALA/SFC improved the glucose tolerance in overnight fasted mice (Fig. 4e). Following 5 h fasting, the plasma glucose concentrations of ALA/SFC administered DIO mice and ND fed mice were almost the same as measured by OGTT. Taken together, there data suggest that ALA/SFC improves glucose tolerance.

### ALA/SFC enhances glucose uptake in 3T3-L1 adipocytes and L6 myotubes

**Fig. 5:**
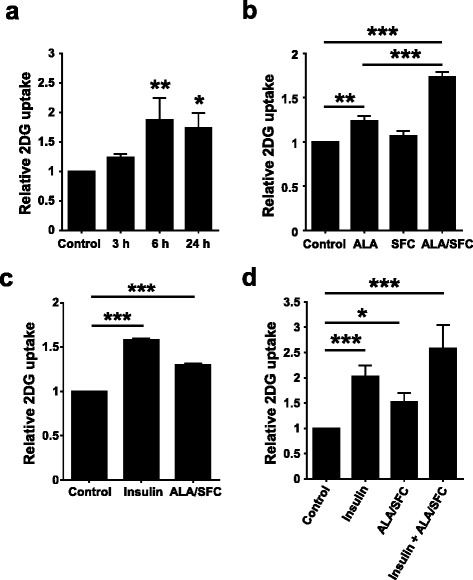
ALA/SFC induces glucose uptake in cells. Differentiated 3T3-L1 or L6 cells were incubated in KRPH buffer for 18 min at 37 °C and then further incubated with KRPH buffer containing 1 mM 2DG with or without insulin for 20 min. Results are shown as means ± SD relative to the 2DG uptake of controls (set to 1.0). **a** The effect of treatment of ALA (0.5 mM)/SFC (0.25 mM) on glucose uptake for the indicated times in 3T3-L1 adipocytes. *p < 0.05, **p < 0.01, vs. control (Dunnett’s test, *n* = 3). **b** The effect of only ALA (0.5 mM), SFC (0.25 mM), or ALA (0.5 mM)/SFC (0.25 mM) treatment for 6 h on glucose uptake in 3T3-L1 adipocytes. **p < 0.01, ***p < 0.001 vs. control (Bonferroni’s test, *n* = 3). **c** The effect of treatment of ALA (0.5 mM)/SFC (0.25 mM) on glucose uptake for 6 h in L6 myotubes. Insulin (100 nM) was used as a positive control. ***p < 0.001 vs. control (Dunnett’s test, *n* = 3). **d** The effect of ALA (0.5 mM)/SFC (0.25 mM) treatment on insulin action to glucose uptake in 3T3-L1 adipocytes. The concentration of insulin was 10 nM. *p < 0.05, ***p < 0.001 vs. control (Dunnett’s test, *n* = 4)

As ALA/SFC improves glucose tolerance as shown in Fig. 4, we considered that it might consequently directly enhance glucose uptake in cells. To confirm this, the effects of ALA/SFC on glucose uptake in 3T3-L1 adipocytes and L6 myotubes were examined. We first focused on 3T3-L1 adipocytes because ALA/SFC reduced their lipid content. At 6 h glucose uptake was significantly enhanced by approximately 90% (Fig. 5a). The glucose uptake by treatment with ALA alone was enhanced by 23%. This effect was weaker than that of ALA/SFC treatment (Fig. 5b), but treatment with SFC alone showed no enhancement of glucose uptake.

In L6 skeletal cells, ALA/SFC also significantly enhanced glucose uptake by 30% (Fig. 5c), which was consistent with the results shown in Fig. 5a and b. Treatment of L6 skeletal cells with ALA/SFC did not induce cytotoxic effect under the conditions used. In addition, neither ALA nor SFC alone enhanced glucose uptake in L6 skeletal cells (data not shown). Taken together, these data suggest that ALA combined with ferrous ion enhances glucose uptake in vitro.

We also examined whether ALA/SFC enhances the glucose uptake stimulated by insulin in 3T3-L1 cells. Treatment of insulin, ALA/SFC, and ALA/SFC combined with insulin enhanced glucose uptake by about 2, 1.5, and 2.6-fold, respectively (Fig. 5d). These data suggest that ALA/SFC has an additive effect on insulin stimulation, but does not have a synergistic effect in 3T3-L1 cells.

### ALA/SFC enhances the expression levels of mitochondrial OXPHOS complexes III, IV, and V in WAT of DIO mice

**Fig. 6:**
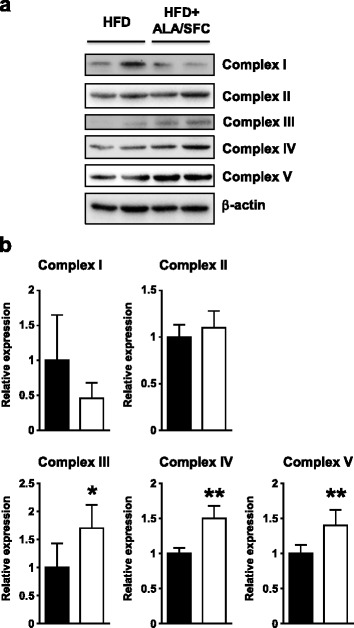
Expression levels of mitochondrial complexes I–V in epididymal WAT of DIO mice. **a** The expression levels of complex I–V proteins in WAT were analyzed by immunoblotting. **b** The relative expression levels of complexes I–V to β-actin. The expression levels of ALA/SFC-administered mice are shown as relative values compared to those of the vehicle-administered DIO mice. *p < 0.05, **p < 0.01 vs. vehicle-administered DIO mice (Student’s *t*-test, *n* = 5)

ALA upregulates not only the expression of mitochondrial OXPHOS complex IV, but also ATP production in the liver of mouse [19]. Thus, ALA/SFC might upregulate the expression of the mitochondrial OXPHOS complex IV in DIO mouse WAT as well. We examined the expression levels of not only mitochondrial OXPHOS complex IV but also of other mitochondrial complexes in epididymal WAT. The expression levels of complex III, IV, and V in ALA/SFC-administered DIO mice were about 40–70% higher than those in vehicle-administered DIO mice (Fig. 6). However, the expression levels of complex I and II were not significantly different between ALA/SFC-administered and vehicle-administered DIO mice. These data suggest that ALA/SFC enhances the expression levels of not only mitochondrial OXPHOS complex IV, but also complexes III and V in the WAT of DIO mice.

## Discussion

This study examined the effects of ALA/SFC on obesity and glucose intolerance in DIO mice. ALA/SFC administration yielded a reduction of body weight, especially in the weight of epididymal and visceral WAT in DIO mice. ALA/SFC also resulted in a reduction of lipid content in 3T3-L1 adipocytes. Furthermore, ALA/SFC treatment improved glucose tolerance in DIO mice. In both 3T3-L1 adipocytes and L6 skeletal muscle cells, ALA/SFC enhanced glucose uptake. Finally, ALA/SFC increased the expression levels of mitochondrial complex III, IV, and V in WAT in DIO mice. Our results are consistent with previous reports demonstrating that ALA regulates glucose and/or lipid metabolism [18, 32–36] and thus provide additional evidence that ALA/SFC administration can improve glucose and/or lipid metabolism.

This study found that ALA/SFC enhanced glucose uptake and reduced lipid content in 3T3-L1 adipocytes. In contrast, treatment with ALA alone caused only weak glucose uptake enhancement and lipid content reduction, whereas SFC alone did not affect glucose uptake or lipid content. These data indicate that the combination of ALA and ferrous ions is important to enhance glucose uptake and reduce lipid content in 3T3-L1 adipocytes. Notably, heme is generated by the insertion of a ferrous ion into PpIX, which is synthesized from 8 molecules of ALA [13]. ALA combined with ferrous ion has been shown to enhance heme production in vitro in cultures of bovine pulmonary arteries [21], RAW264 macrophage cell lines [22], and the human gastric cancer cell line MKN28 [23]. Thus, an increase of heme production might be important to enhance glucose uptake and reduce lipid content. Several of the mitochondrial proteins that compose OXPHOS contain heme [37], and heme deficiency is associated with the selective decrease of activity and protein expression of complex IV [15]. Accordingly, the increased heme production mediated by ALA/SFC might lead to a concomitant increase of complex IV expression and activity. In fact, our findings show that ALA/SFC upregulates the expression level of mitochondrial OXPHOS complex IV in WAT of DIO mice. Additionally, although complex V does not contain heme, ALA/SFC also upregulates the expression levels of complexes III and V in WAT. Ogura et al. [19] have reported that ALA upregulates mitochondrial complex IV expression and activity in addition to enhancing aerobic energy metabolism in the liver of normal mice [19]. Taken together, these studies suggest that ALA/SFC can improve mitochondrial function in adipocytes and muscle, thereby increasing energy metabolism. This hypothesis is supported by the report that the administration of low-dose ALA for 14 days in Sprague Dawley rats fed with ND increases the oxygen consumption, decreases the weight of WAT, and induces expression of UCP1 in brown adipose tissue [18]. The enhancement of energy metabolism can upregulate lipid and glucose consumption, which might lead to a reduction of adiposity and enhancement of glucose uptake, respectively. In turn, it is likely that increased glucose uptake by ALA/SFC can improve glucose tolerance. In addition, the enhancement of energy metabolism would increase the production of some metabolites such as NAD+ and ATP. NAD+ activates Sirt 1 that plays an essential role in glucose and lipid homeostasis [38, 39]. Thus, ALA/SFC might upregulate Sirt 1 signaling pathway, leading to the improvement of glucose and/or lipid metabolism. However, the detailed molecular mechanisms concerning how ALA/SFC reduces lipid content, enhances glucose uptake, and induces the expression of mitochondrial complexes III, IV, and V remain to be elucidated.

It is well known that insulin plays an essential role in glucose uptake in adipocytes and muscle cells [40]. However, we found that ALA/SFC did not enhance insulin action on glucose uptake in adipocytes. Because Akt plays an important role in the insulin signaling pathway [40], we investigated the phosphorylation levels of Akt following treatment. However, the levels of Akt phosphorylation in ALA/SFC-treated 3T3-L1 adipocytes were not different from those in vehicle-treated cells (data not shown). In addition, in this study the plasma insulin levels of ALA/SFC-administrated DIO mice were not different from those of vehicle-DIO mice (data not shown). Furthermore, our previous report shows that in OGTT, the plasma insulin levels of ZDF rats administered 300/47.1 mg/kg ALA/SFC were not significantly different from those of the rats administered the vehicle [36]. Higashikawa et al. [33] reported that the fasting insulin levels in pre-diabetes patients were not affected when ALA/SFC was administered for 12 weeks although the glucose tolerance was improved [33]. Together, these findings suggest that a glucose-lowering effect by ALA/SFC might be mediated by insulin-independent mechanisms.

## Conclusions

Our findings revealed that ALA/SFC reduces adiposity and improves glucose tolerance in DIO mice, and suggested that these improvements are in part the consequence of an improvement of mitochondrial function. However, further studies are required to elucidate the molecular mechanisms underlying the upregulation of mitochondrial function by ALA/SFC. Our results suggest that ALA/SFC might be a useful supplement to combat obesity and obesity-related metabolic disease such as T2DM and cardiovascular disease.

## Additional file

Additional file 1: Raw data for **Figures 2 to 6**. (XLSX 39 kb)

## Abbreviations

2DG: 2-deoxyglucose
ALA: 5-aminolevulinic acid
AUC: Area under the curve
DIO: Diet-induced obesity
HFD: High-fat diet
KRPH: Krebs-Ringer’s phosphate
ND: Normal diet
OGTT: Oral glucose tolerance test
OXPHOS: Oxidative phosphorylation
PpIX: Protoporphyrin IX
SFC: Sodium ferrous citrate
T2DM: Type 2 diabetes mellitus
WAT: White adipose tissue
